# Indirect treatment comparison of oral sebetralstat and intravenous recombinant human C1 esterase inhibitor for on-demand treatment of hereditary angioedema attacks

**DOI:** 10.1186/s13223-025-00955-6

**Published:** 2025-03-15

**Authors:** H. Henry Li, Emel Aygören-Pürsün, Markus Magerl, Timothy J. Craig, Michael E. Manning, Noemi Hummel, Agnieszka Kopiec, Shuai Fu, James Morris, Alice Wang, Paul K. Audhya, Jonathan A. Bernstein

**Affiliations:** 1 Institute for Asthma and Allergy, Chevy Chase, MD USA; 2 University Hospital Frankfurt, Goethe University, Frankfurt, Germany; 3 Angioedema Center of Reference and Excellence (ACARE), Institute of Allergology, Charité – Universitätsmedizin Berlin, corporate member of Freie Universität Berlin and Humboldt-Universität Zu Berlin, Berlin, Germany; 4 Fraunhofer Institute for Translational Medicine and Pharmacology ITMP, Immunology and Allergology, Berlin, Germany; 5 Penn State University, Hershey, PA USA; 6 Vinmec International Hospital, Times City, Hanoi, Vietnam; 7 Asthma and Immunology Associates, Ltd, Scottsdale, AZ USA; 8 University of Arizona College of Medicine-Phoenix, Scottsdale, AZ USA; 9 Certara, Lörrach, Germany; 10 Certara, Krakow, Poland; 11 Certara, Shanghai, China; 12 Cogentia, Cambridge, UK; 13 KalVista Pharmaceuticals, Cambridge, MA USA; 14 University of Cincinnati, College of Medicine, Cincinnati, OH USA; 15 Bernstein Clinical Research Center, Cincinnati, OH USA

**Keywords:** Hereditary angioedema, Indirect treatment comparison, Matching-adjusted indirect comparison, rhC1INH, Sebetralstat

## Abstract

**Background:**

The goal of on-demand treatment for hereditary angioedema attacks is to halt attack progression to minimize morbidity and mortality. Four on-demand treatments have been approved thus far (ecallantide, icatibant, recombinant human C1 esterase inhibitor [rhC1INH], and plasma-derived C1INH). Results from the sebetralstat phase 3 KONFIDENT trial (NCT05259917) have been reported. To put these results into context without head-to-head trials, an indirect treatment comparison (ITC) was conducted to facilitate comparisons of efficacy and safety across treatment options.

**Methods:**

Based on a systematic literature review and feasibility assessment, only the pivotal trial for intravenous rhC1INH (NCT01188564) reported necessary data for a comparable primary efficacy endpoint (time to beginning of symptom relief) to enable an ITC with oral sebetralstat. Bayesian fixed-effects network meta-analyses models were conducted to indirectly compare the efficacy and safety outcomes of sebetralstat and rhC1INH (NCT01188564, NCT00225147, NCT00262301). A matching-adjusted indirect comparison (MAIC) of efficacy was performed, adjusting for baseline attack severity and demographic characteristics.

**Results:**

The fixed-effects model found no significant differences in time to beginning of symptom relief between sebetralstat 300 mg and rhC1INH 50 IU/kg (hazard ratio [95% credible interval], 0.96 [0.42–2.15] to 1.19 [0.58–2.45]). After adjusting for baseline attack severity, the MAIC showed numerically favorable results with sebetralstat compared with rhC1INH, regardless of whether baseline demographics were matched. The fixed-effects model found no significant differences in treatment-related treatment-emergent adverse events. All sensitivity analyses returned consistent results.

**Conclusions:**

This ITC found no significant differences in time to beginning of symptom relief and overall treatment-related treatment-emergent adverse events between sebetralstat and rhC1INH.

**Supplementary Information:**

The online version contains supplementary material available at 10.1186/s13223-025-00955-6.

## Background

Hereditary angioedema (HAE), a rare, autosomal dominant disorder caused by mutations in the C1 inhibitor (C1INH) gene, is characterized by painful, episodic subcutaneous or mucosal swelling of the extremities, trunk, face, genitalia, or larynx [1–3]. C1INH inactivates components of the kallikrein–kinin (contact) system, which mediates angioedema [1–4]. Patients with HAE are affected most often by cutaneous attacks, but more than 50% will experience at least one laryngeal attack, which can be fatal if left untreated [1, 3]. Because of the unpredictable nature of attacks, guidelines recommend that patients have ready access to on-demand treatment, which can be administered early to halt attack progression [5–7]. The goal of on-demand treatment is to quickly inhibit the contact system cascade and minimize fluid extravasation during an attack [4–7].

Four on-demand treatments have been approved globally: plasma-derived C1 esterase inhibitor concentrate (pdC1INH), icatibant (bradykinin B2 receptor antagonist), ecallantide (plasma kallikrein inhibitor approved in the US), and recombinant human C1 esterase inhibitor concentrate (rhC1INH). As on-demand treatments, pdC1INH and rhC1INH are infused intravenously, whereas icatibant and ecallantide are injected subcutaneously. C1INH concentrates and icatibant can be self-administered, but due to the risk of anaphylaxis, ecallantide can only be administered by a health care professional (HCP) in a monitored setting [5–7]. Although HAE guidelines have been updated in recent years, encouraging the early use of on-demand treatment [5–7], it has been more than a decade since the last on-demand treatment was approved by a regulatory authority. None of the guidelines designate any of these as a preferred treatment [5–7].

Sebetralstat is an investigational plasma kallikrein inhibitor for the on-demand treatment of HAE attacks [8]. In contrast to currently approved on-demand therapies, which are administered parenterally, sebetralstat is administered orally [9]. In the phase 3 KONFIDENT trial (NCT05259917), compared with placebo, sebetralstat (300 or 600 mg) was associated with faster times to the beginning of symptom relief, reduction in attack severity, and complete attack resolution, with a similar safety profile and no serious adverse events (AEs) [8].

To support clinical decision-making, it would be desirable to compare the efficacy and safety profile of sebetralstat with those of other approved treatments. In the absence of head-to-head trials, an indirect treatment comparison (ITC) represents an internationally recognized and valid statistical methodology [10] and, in this case, the only approach to compare clinical outcomes of various on-demand treatments. However, to conduct an ITC, the trial designs and patient populations of trials must be comparable, as determined by a feasibility assessment [11]. To date, ITCs in the HAE setting have focused on prophylactic treatment [12–15]; none have been published for on-demand treatments. This ITC was conducted to compare sebetralstat with other on-demand treatments for HAE attacks using publicly available data from phase 3 trials.

## Methods

### Systematic literature review

To identify trials to include in the ITC, a systematic literature review (SLR) was performed using the data selection process followed the Centre for Reviews and Dissemination (CRD) guidelines and Cochrane methodology [16, 17]. The scope of the SLR reflected predefined eligibility criteria that followed the Population, Intervention, Comparators, and Outcomes (PICO) criteria (Supplementary Table 1). Bibliographic details and abstracts of all citations retrieved from the literature search were imported into EndNote (Berkeley, CA, US), a citation management software program, to allow de-duplication prior to screening. After deduplication, records were imported into Rayyan (Cambridge, MA, US), an abstract screening tool. Two independent reviewers conducted the initial screening based on titles and abstracts, followed by the second screening of full-text articles. Any discrepancies between reviewers at either screening were resolved by a third independent reviewer. Data extraction of the included trials was undertaken in Microsoft Excel (Microsoft Corp., Redmond, WA, USA) by one reviewer, and the second reviewer checked the extracted data, and discrepancies between trial data and extracted data were resolved. In cases in which more than one publication described a trial, the data were compiled into a single entry in the data extraction table to avoid double-counting of participants and trials. The SLR was reported according to the Preferred Reporting Items for Systematic Review and Meta-Analysis (PRISMA) guidelines [18] (Supplemental Fig. 1). The SLR included a review of “grey” literature to identify data from sources not always indexed in the electronic databases but available from scientific conferences. Modified versions of the terms used for the database searches were used to search the following grey literature sources: European Academy of Allergy and Clinical Immunology (EAACI), American Academy of Allergy, Asthma and Immunology (AAAAI), American College of Allergy, Asthma & Immunology (ACAAI), Academy of Managed Care Pharmacy (AMCP), and ISPOR—The Professional Society for Health Economics and Outcomes Research.

### ITC feasibility assessment

A feasibility assessment was conducted to determine which trials identified in the SLR met the criteria for inclusion in the ITC. Reporting of outcomes and the similarity of definitions of outcomes were assessed. Baseline demographics and disease characteristics were compared across trials, and levels of redosing, rescue, and concomitant medications were assessed for similarity (Supplementary Table 2). Trials were eligible for inclusion if they did not differ substantially with respect to outcome definitions, reported data on comparable outcomes, relevant statistical outputs, follow-up times, patient characteristics, use of rescue/concomitant medication, and level of connectedness of evidence networks for the final endpoint. Based on the feasibility assessment, trials with comparable endpoints were included in the ITC.

### Statistical methods

Across statistical analyses, this ITC used patient-level data from the KONFIDENT trial and aggregate data from the other included trials to compare on-demand treatments of HAE. Network meta-analyses (fixed-effects or random-effects models) were used to compare the following endpoints, which were selected based on the results of the feasibility assessment: (1) time to beginning of symptom relief and (2) incidence of treatment-related treatment-emergent AEs (TEAEs). For the purpose of this study, both direct and indirect evidence—including from the placebo arms—were used in these analyses. The network meta-analysis methodology is an accepted statistical technique that allows estimation of the relative effects of treatments across different trials by using a common comparator between the trials [19–23].

Bayesian fixed-effects and random-effects models were used to compare time to beginning of symptom relief, based on hazard ratios (HRs) and corresponding credible intervals (CrIs). For the efficacy analysis, fixed-effects meta-analyses with inverse variance weights were applied to obtain single HRs from two stratifications (region: US and non-US, sex: female and male) for the comparator study. Bayesian fixed-effects and random-effects models were also used to compare treatment-related TEAEs, with comparisons based on odds ratios (ORs) and corresponding CrIs. The random-effects models served as a sensitivity analysis for determining if the fixed-effects models (base-case analysis) were appropriate for use as the main analysis based on similar deviance information criterion (DIC).

Matching-adjusted indirect comparisons (MAICs) of time to beginning of symptom relief were conducted, as differences in baseline disease severity and demographics may have affected time to beginning of symptom relief. The MAICs included two scenarios: Scenario 1 adjusted for baseline severity only and Scenario 2 adjusted for both baseline severity and demographics (age, sex, and race) as matching variables. Matching baseline patient characteristics were used to enhance the comparability between the heterogeneous trials. HRs and corresponding confidence intervals (CIs) of MAICs for the time to beginning of symptom relief were calculated for the comparison of sebetralstat 300 mg versus rhC1INH 50 IU/kg. MAICs support robust ITCs by using propensity score weighting to improve comparability between trials and adjusting for cross-trial population differences [24].

## Results

### SLR

The SLR identified 15 randomized controlled trials (RCTs), four open-label extension trials, and two non-randomized trials, with a total of 68 reports (Supplemental Table 3).

### Feasibility assessment

Thirteen trials were included in the feasibility assessment. Based on the feasibility assessment, differences observed across trial designs resulted in most trials being excluded. Differences in trial design included variations in definitions and measurement of time to beginning of symptom relief (e.g., using visual analog scale [VAS] vs Patient Global Impression of Change [PGI-C] scale; Supplementary Table 4); time to study-drug administration, use of rescue medication, and censoring (Supplementary Table 5); and AE reporting (e.g., reporting AEs or TEAEs vs treatment-related TEAEs; Supplementary Table 6).

**Fig. 1:**
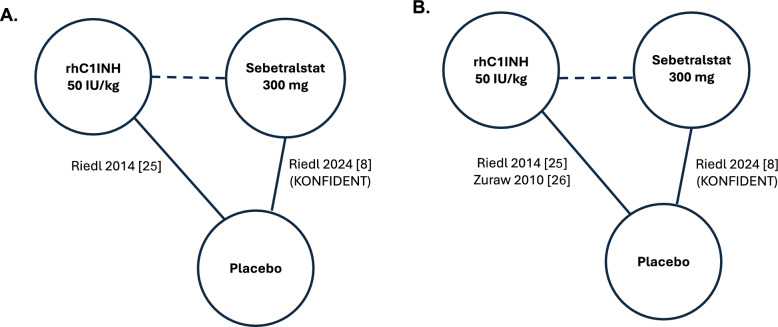
Evidence base for the indirect treatment comparison on **A** symptom relief and **B** treatment-related treatment-emergent adverse event. rhC1INH, recombinant human C1 esterase inhibitor

For the primary endpoint of time to beginning of symptom relief, only the phase 3 rhC1INH C1-1310 trial (rhC1INH 50 IU/kg vs placebo) [25] used a measure comparable to the Patient Global Impression of Change (PGI-C) scale in the phase 3 KONFIDENT trial (sebetralstat 300 mg vs placebo) [8], and thus, was deemed to be appropriate for inclusion in the indirect comparison and the MAICs (Fig. 1A). For the safety comparison, the phase 3 KONFIDENT trial (sebetralstat 300 mg vs placebo) [8] reported only treatment-related TEAEs. As such, only three other trials reporting this measure were deemed to be appropriate for inclusion in the indirect comparison for: (1) the phase 3 rhC1INH C1-1310 trial (rhC1INH 50 IU/kg vs placebo) [25], and the pooled analysis of (2) the phase 2/3 rhC1INH C1-1205–01 trial (rhC1INH 50 IU/kg vs placebo) and (3) the phase 3 C1-3401–01 trial (rhC1INH 100 IU/kg vs placebo [26]; Fig. 1B).

Most baseline demographics were comparable across the four trials included in the ITC (Table 1) [8, 25, 26]. Most patients were White (range, 84%–100%) and female (range, 56%–92%), with mean or median ages of approximately 40 years. There was a numerical difference between percentages of patients receiving long-term prophylactic (LTP) therapy in the interventional groups in the KONFIDENT and rhC1INH C1-1310 trials (22% vs 50%, respectively) [8, 25].

**Table 1:** Baseline demographics and clinical characteristics of patients in the clinical trials included in the ITCs^a^

	KONFIDENT [8]		C1-1310 [25]		C1-1205–01/C1-3401–01 [26]		
	Oral sebetralstat 300 mg (*n* = 87)	Oral placebo (*n* = 84)	Intravenous rhC1INH 50 IU/kg (*n* = 44)	Intravenous placebo (*n* = 31)	Intravenous rhC1INH 50 IU/kg (*n* = 12)^b^	Intravenous placebo (*n* = 13)^b^	Intravenous placebo (*n* = 16)^c^
**White,** ***n*** **(%)**	73 (84)	73 (87)	42 (95)	30 (97)	12 (100)	11 (85)	16 (100)
**Female,** ***n*** **(%)**	54 (62)	55 (65)	28 (64)	19 (61)	8 (67)	12 (92)	9 (56)
**Age, y**							
Mean (SD)	NR	NR	39.4 (12.59)	41.4 (15.38)	40.7 (12.2)	32.4 (11.3)	44.5 (16.8)
Median (IQR)	37.0 (25.0–49.0)	38.0 (25.0–49.0)	NR	NR	NR	NR	NR
**Use of long-term prophylactic treatment,** ***n*** **(%)**	19 (21)	18 (22)	22 (50)	15 (48)	NR	NR	NR
**Baseline severity,** ***n*** **(%)**							
None	0	2 (2.4)	—	—	—	—	—
Mild	36 (41)	36 (43)	—	—	—	—	—
Moderate	35 (40)	33 (39)	—	—	—	—	—
Severe	12 (14)	10 (12)	44 (100)	31 (100)	12 (100)	31 (100)	16 (100)
Very severe	2 (2)	3 (4)	—	—	—	—	—
Missing	2 (2)	0	—	—	—	—	—

^a^KONFIDENT and C1-1310 were included in the ITC of efficacy (time to beginning of symptom relief). Data from KONFIDENT, C1-1310, and a pooled analysis of C1-1205–01 and C1-3401–01 were included in the ITC of safety (incidence of treatment-related treatment-emergent adverse events)

^b^Data from C1-1205–01

^C^Data from C1-3401–01

IQR, interquartile range; ITC, indirect treatment comparison; rhC1INH, recombinant human C1 esterase inhibitor; NR, not reported; SD, standard deviation

Although definitions for time to beginning of symptom relief were comparable between the trials (Table 2), there were other notable differences [8, 25]. Time to beginning of symptom relief was measured using the PGI-C scale in the KONFIDENT trial and the Treatment Effect Questionnaire (TEQ) in the rhC1INH C1-1310 trial. In the KONFIDENT trial, patients were instructed to treat as early as possible at home after the onset of the attack, regardless of severity, as measured by the Patient Global Impression of Severity scale. In contrast, in the rhC1INH C1-1310 trial, patients were eligible for treatment if the onset of their attack occurred within 5 h before presentation to the clinical trial site and the patient-assessed Overall Severity VAS score (0–100-mm scale) was at least 50 mm at presentation and just before dosing by the HCP. Furthermore, the follow-up time for the primary endpoint was 12 h in the KONFIDENT trial and 24 h in the rhC1INH C1-1310 trial, and use of rescue therapy differed between the two trials (Supplementary Table 5). HRs for time to beginning of symptom relief were not provided in the published report for the rhC1INH C1-1310 trial [25]; however, the US Food and Drug Administration publicly available prescribing information for rhC1INH includes HRs for region (US and non-US) and sex (female and male) [27], that were subsequently combined into single HRs using meta-analysis.

**Table 2:** Primary endpoint definition of time to the beginning of symptom relief

Trial	Time to symptom relief definition	Time to symptom relief tool	Questions	Response options	Endpoint achieved
KONFIDENT [8]	Beginning of symptom relief	PGI-C	How would you describe your overall HAE attack symptoms right now, compared to how you were when you took the trial medication?	• Much worse • Worse • A little worse • No change • A little better • Better • Much better	When a rating of at least “a little better” on the PGI-C scale for at least two time points in a row within 12 hours after first dose of study drug
rhC1INH [25]	Time to onset of sustained relief	TEQ	Q1: To what extent has the Overall Severity of your [attack location] HAE attack changed since you received the infusion?	• Much worse • Worse • A little worse • No change • A little better • Better • Much better	Time between dosing and first assessment when patient answered, “a little better,” “better,” or “much better” for Q1; answered “yes” for Q2; and persistence of improvement at next assessment (i.e., either the same or a better response to Q1 and “yes” to Q2), with follow-up over a 24-hour period
			Q2: Overall, has the intensity of your [relevant attack location] HAE attack symptoms begun to decrease noticeably since you received the infusion?	• Yes • No	

HAE, hereditary angioedema; PGI-C, Patient Global Impression of Change; Q, question; TEQ, Treatment Effect Questionnaire

### Bayesian fixed-effects model for time to beginning of symptom relief

**Fig. 2:**
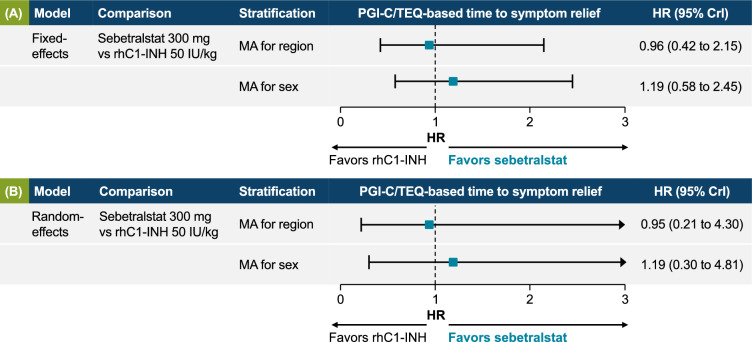
Time to beginning of symptom relief per **A** fixed-effects (base case) and **B** random-effects (sensitivity analysis) models. Hazard ratio (HR) values > 1 favor sebetralstat 300 mg over recombinant human C1 esterase inhibitor (rhC1INH) 50 IU/kg. *CrI* credible interval, *MA* meta-analysis, *PGI-C* Patient Global Impression of Change, *TEQ* Treatment Effect Questionnaire

The fixed-effects model found no significant difference in time to beginning of symptom relief between sebetralstat 300 mg and rhC1INH 50 IU/kg in the fixed-effects models for region (HR, 0.96; 95% CrI, 0.42–2.15; Fig. 2A) or sex (HR, 1.19; 95% CrI, 0.58–2.45; Fig. 2B), although time to beginning of symptom relief numerically favored sebetralstat in the meta-analysis for sex. No differences were observed in the random-effects models (sensitivity analysis) for region (HR, 0.95; 95% CrI, 0.21–4.30) or sex (HR, 1.19; 95% CrI, 0.30–4.81). Given the simplicity of the indirect comparison for time to beginning of symptom relief (only two comparisons from two trials) and similar DIC for the fixed-effects and random-effects models (Supplementary Table 7), the fixed-effects model was considered appropriate as the main analysis. Inputs for the fixed-effects model for time to beginning of symptom relief are shown in Supplementary Table 8.

### MAIC for time to beginning of symptom relief

**Fig. 3:**
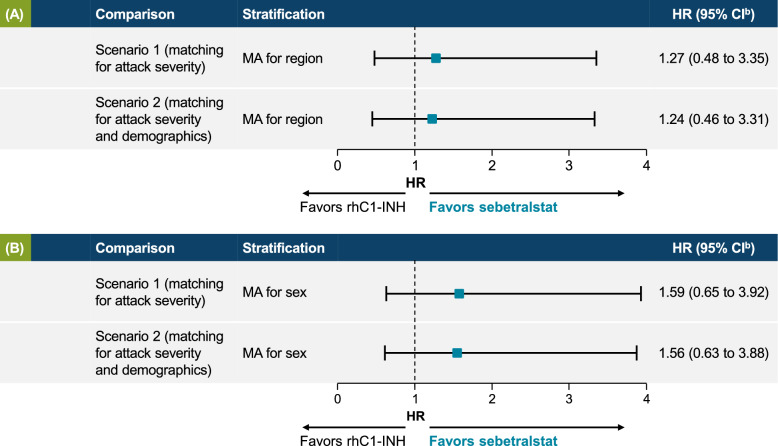
Time to beginning of symptom relief matched for **A** baseline attack severity^a^ only and **B** baseline attack severity,^a^ age, sex, and race. ^a^Maximum of three baseline Overall Severity visual analog scale (VAS) scores. ^b^Hazard ratio (HR) values > 1 favor sebetralstat 300 mg over rhC1INH 50 IU/kg. CI, confidence interval; rhC1INH, recombinant human C1 esterase inhibitor; MA, meta-analysis; MAIC, matching-adjusted indirect comparison

Two MAICs were performed under each of the two match-adjustment scenarios (baseline severity only and baseline attack severity plus demographics) because HRs were only available for region and sex in the rhC1INH C1-1310 trial [27]. The MAICs found no significant difference in time to beginning of symptom relief between sebetralstat 300 mg and rhC1INH 50 IU/kg in either scenario. In Scenario 1, after matching for baseline attack severity, time to beginning of symptom relief numerically favored sebetralstat 300 mg versus rhC1INH 50 IU/kg (region: HR, 1.27 [95% CI, 0.48–3.35]; sex: HR, 1.59 [95% CI, 0.65–3.92]; Fig. 3A). In Scenario 2, after matching for baseline severity and patient demographics, the results did not appreciably change (region: HR, 1.24 [95% CI, 0.46–3.31]; sex: HR, 1.56 [95% CI, 0.63–3.88]; Fig. 3B).

### Bayesian fixed-effects model for treatment-related TEAEs

**Fig. 4:**
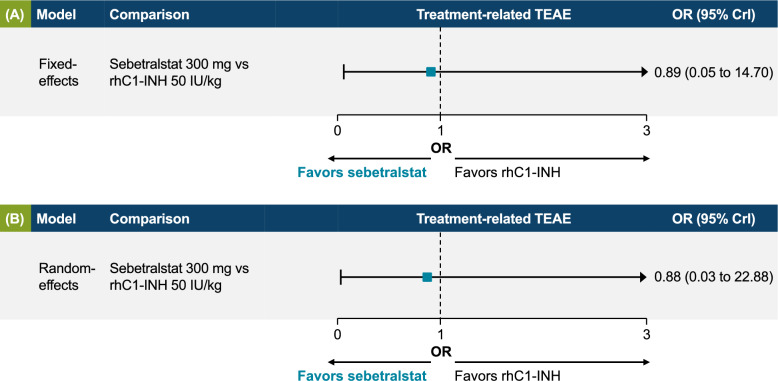
Treatment-related treatment-emergent adverse events (TEAEs) per **A** fixed-effects (base case) and **B** random-effects (sensitivity analysis) models. Odds ratio (OR) values < 1 favor sebetralstat 300 mg over rhC1INH, recombinant human C1 esterase inhibitor (rhC1INH) 50 IU/kg. *CrI* credible interval

The safety fixed-effects model found no significant difference in treatment-related TEAEs between sebetralstat 300 mg and rhC1INH 50 IU/kg in the fixed-effects model (OR, 0.89; 95% CrI, 0.05–14.70; Fig. 4A) or the random-effects model (sensitivity analysis; OR, 0.88; 95% CrI, 0.03–22.88; Fig. 4B). Given the simplicity of the indirect comparison for treatment-related TEAEs (only two comparisons from four trials) and similar DIC for the fixed-effects and random-effects models (Supplementary Table 9), the fixed-effects model was considered appropriate as the main analysis. Inputs for the fixed-effects model for treatment-related TEAEs are shown in Supplementary Table 10.

## Discussion

This ITC—the first to successfully compare on-demand treatments in HAE—evaluated findings from the KONFIDENT trial of sebetralstat in the context of currently available on-demand treatments. Indirect comparisons of on-demand treatments have been challenging due to the heterogeneity in trial designs and outcomes. Indeed, results of a systematic review of 13 on-demand treatment trials revealed the use of 72 different standardized efficacy outcome terms, none of which was reported consistently across all trials [28]. An attempt was made by Bork et al. to conduct an ITC of on-demand treatments for laryngeal attacks, but because of the heterogeneity in efficacy endpoints, the researchers were ultimately able to undertake only a descriptive comparison [29].

Because the KONFIDENT trial protocol reflected changes in treatment guidelines, which now advocate for the early treatment of all attacks [5–7], its design was distinct from the pivotal phase 3 trials of currently approved on-demand treatments [30]. As such, our feasibility assessment resulted in the exclusion of trials for 3 of the 4 currently approved on-demand treatments. Assessment of the designs and outcome measures (Supplementary Tables 4–6) of the IMPACT 1 trial of pdC1INH [31], the EDEMA3 and EDEMA4 trials of ecallantide [32, 33], the FAST-1 and FAST-3 trials of icatibant [34, 35], and the two phase 3 trials of nanofiltered pdC1INH [36] revealed that none used a primary endpoint measure comparable with the PGI-C scale used in the KONFIDENT trial. A core set of efficacy measures for HAE trials may facilitate more robust ITCs in the future [37], such as the recently initiated phase 3 trial RAPIDe-3, which is evaluating efficacy of oral deucrictibant using the PGI-C scale, however, whether patients are instructed to treat attacks as early as possible is currently unknown [38]. Approaches to the use, type, and timing of rescue medication and to the censoring of patients also varied across trials (particularly in FAST-1 and FAST-3 versus KONFIDENT); the potential influence of these trial characteristics on efficacy outcomes further impeded the inclusion of these trials in the ITC (Supplementary Table 5). Finally, although HRs for the primary endpoint comparing sebetralstat with placebo were available, neither HRs nor Kaplan–Meier curves were published for any of these pivotal trials, with the exception of Zuraw et al. [36], which reported results from two trials of nanofiltered pdC1INH.

Based on the feasibility assessment, in terms of efficacy, the KONFIDENT trial of sebetralstat could only be compared with one rhC1INH trial. Although a comparison of sebetralstat with rhC1INH was deemed feasible, it should be noted that KONFIDENT used the PGI-C scale to measure time to beginning of symptom relief, whereas the trial of rhC1INH used the TEQ (Table 2). Despite differences in routes of administration, mechanisms of action, and trial designs, the efficacy of sebetralstat was comparable with that of rhC1INH. Because differences in baseline attack severity between the two trials could have affected the time to beginning of symptom relief, we also performed MAICs. After adjusting for differences in baseline severity, sebetralstat was associated with a numerically faster time to beginning of symptom relief compared with rhC1INH. Use of LTP was permitted in both trials. Although the percentage use was higher in the rhC1INH trial than in the KONFIDENT trial (50% vs 22%) [8, 25], a systematic review found that (1) a substantial proportion of patients (> 55%) using LTP did not achieve attack-free status and (2) there were insufficient data to suggest LTP directly caused a reduction in attack severity [39]. Thus, despite differences in baseline attack severity and the proportion of patients using LTP, the efficacy of sebetralstat and rhC1INH was shown to be comparable in this ITC, a finding that was not unexpected given similar pharmacokinetics (i.e., rapid adsorption and distribution) and physiologic effects (i.e., rapid interdiction of the contact system). Along with efficacy, no significant differences in safety were found (excluding injection-site reactions with rhC1INH) between sebetralstat and rhC1INH.

This ITC compared efficacy and safety data derived from the RCT setting. However, RCTs may not accurately reflect real-world use of the currently available parenterally administered on-demand treatments. Surveys of patients with HAE have highlighted both positive and negative factors associated with real-world treatment practices. The results of one survey revealed consequences of the complex decision-making process patients face while self-administering on-demand treatment; patients frequently delayed treatment or did not treat attacks due to injection-site reactions, “fear of needles”, and concerns about the cost of refilling their prescription [40, 41]. Overcoming these barriers is important to reduce the number of untreated attacks, and prompt use of on-demand treatment may minimize or prevent the development of serious sequalae, such as painful gastrointestinal symptoms and life-threatening laryngeal edema [5–7]. A comparison of median time to treatment in patients receiving HCP-administered icatibant in the FAST-3 trial (post hoc analysis) and in the real-world Icatibant Outcome Survey (IOS) found that patients received icatibant earlier in the real-world setting (6.5 vs 2.0 h, respectively, *P* < 0.001) [42]. Additional analyses of data from IOS found that median time to administration was significantly shorter in self- versus HCP-treated attacks (1.5 vs. 2.4 h; *P* = 0.016) [43]. In the KONFIDENT trial, in which patients were instructed to self-administer as early as possible after attack onset—an approach more aligned with real-world treatment practices—the median time to treatment was 41 min with self-administration of sebetralstat [8].

Although this ITC included only two agents, it still provides insights into how sebetralstat, if approved, may fit into the on-demand treatment landscape. An orally administered agent that is at least as efficacious and tolerable as a currently approved parenterally administered on-demand treatment may reduce the barriers associated with treating HAE attacks reported in the real-world setting [40, 41].

### Limitations

In addition to the limitations inherent to an ITC (e.g., heterogeneity in trial designs and endpoints), the sample sizes for the included trials were limited; matching approaches further reduced the number of sebetralstat-treated patients analyzed. Second, results may have differed if an HR for the primary endpoint for the overall rhC1INH trial population had been available. Third, the location of on-demand treatment administration differed, with the KONFIDENT trial being the first phase 3 trial to allow patients to treat at home; the impact of this difference cannot be assessed. Fourth, the ITC of safety was constrained by the limited amount of such data in the public domain and by variations in how these data were reported across publications. Lastly, it is recognized that the ideal comparison between therapies would be a randomized, double-blind, head-to-head clinical trial (double dummy design). However, in the context of HAE, there are multiple reasons making such an RCT infeasible, including challenges in aligning time to treatment for an oral versus an injectable treatment, and the ability to use a double-blind approach for evaluating oral versus injectable treatments, due to expected skin reactions with the latter.

## Conclusions

This ITC found comparable efficacy (measured using time to beginning of symptom relief) and safety (measured using treatment-related TEAEs) between sebetralstat, an investigational, oral plasma kallikrein inhibitor, and intravenous rhC1INH for the on-demand treatment of HAE attacks. These findings are clinically relevant because an orally administered on-demand treatment that is as efficacious and tolerable as an intravenously infused agent may reduce the barriers (e.g., injection-site reactions, fear of injections) that patients associate with parenterally administered agents and that impede early treatment.

## Supplementary Information

**Additional file 1.**

## Data Availability

No datasets were generated or analysed during the current study.

## Abbreviations

AAAAI: American Academy of Allergy, Asthma and Immunology
ACAAI: American College of Allergy, Asthma & Immunology
AE: Adverse event
AMCP: Academy of Managed Care Pharmacy
C1INH: C1 inhibitor
CI: Confidence interval
CRD: Centre for Reviews and Dissemination
CrI: Credible interval
DIC: Deviance information criterion
EAACI: European Academy of Allergy and Clinical Immunology
HAE: Hereditary angioedema
HCP: Health care professional
HR: Hazard ratio
ITC: Indirect treatment comparison
IOS: Icatibant Outcome Survey
LTP: Long-term prophylactic
MAIC: Matching-adjusted indirect comparison
OR: Odds ratio
pdC1INH: Plasma-derived C1 esterase inhibitor
PGI-C: Patient Global Impression of Change
PICO: Population, Intervention, Comparators, and Outcomes
PRISMA: Preferred Reporting Items for Systematic Review and Meta-Analysis
RCT: Randomized controlled trial
rhC1INH: Recombinant human C1 esterase inhibitor
SLR: Systematic literature review
TEAE: Treatment-emergent adverse event
TEQ: Treatment Effect Questionnaire
VAS: Visual analog scale
