# Supplementary material for: Indirect treatment comparison of oral sebetralstat and intravenous recombinant human C1 esterase inhibitor for on-demand treatment of hereditary angioedema attacks
Source: Allergy Asthma Clin Immunol. 2025 Mar 15;21:10. doi: 10.1186/s13223-025-00955-6 (PMC11909897; doi:10.1186/s13223-025-00955-6)
Supplement: Supplementary file 1 — Additional file 1. [file 13223_2025_955_MOESM1_ESM.docx]

**SUPPLEMENTARY MATERIALS**

**Supplemental Table 1** PICO criteria

| **Criterion** | **Inclusion criteria** | **Exclusion criteria** |
| --- | --- | --- |
| Population | Patients with HAE (types I and II) in any setting | - Patients with normal C1INH - Patients with non-HAE |
| Intervention | - Kallikrein inhibitors: sebetralstat, ecallantide - C1INH: pdC1INH, rhC1INH, pdC1INH-nf - β2 receptor antagonist: icatibant | No intervention of interest evaluated |
| Comparators | Placebo, BSC, any treatment that facilitates an indirect comparison (e.g., androgens) | No comparator of interest evaluated |
| Outcomes | - Efficacy: time to treatment, time to the beginning of symptom relief, time to minimal symptoms or almost complete symptom relief, time to complete resolution, redosing rate, use of rescue medication - Safety: AEs by grade; drug-related AEs, type and severity; discontinuation due to AEs | N/A |
| Trial design | - RCTs and OLEs - SLRs and meta-analyses of RCTs (for hand-searching of references only) | Observational studies; retrospective studies; case series/case studies; editorials, commentary, letters, narrative reviews; PK or PD studies; animal or in vitro studies |
| Publication date^a^ | 2007 to date | Prior to 2007 |

AE, adverse event; BSC, best supportive care; C1INH, C1 esterase inhibitor; HAE, hereditary angioedema; IV, intravenous; PD, pharmacodynamics; PK, pharmacokinetics; N/A, not applicable; nf, nanofiltered; OLE, open-label expansion; pd, plasma-derived; PICO, Population, Intervention, Comparators, Outcomes; RCT, randomized controlled trial; rh, recombinant human.

^a^Non-English language publications were excluded.

**Supplemental Table 2** ITC feasibility assessment criteria

| **Feasibility assessment criteria** |
| --- |
| - What outcomes were assessed and reported (e.g., time to the beginning of symptom relief, time to complete resolution, rescue medication use, AEs)? - How similar are the outcome definitions particularly in relation to symptom improvement (e.g., primary symptom VAS 50% reduction or overall symptom change)? - What data were reported based on what follow-up times? - How similar are baseline demographic and disease characteristics across trials? - Was there treatment switching or crossover other than rescue medication? - How similar was rescue/concomitant medication use? - Which trials reported Kaplan–Meier curves for which outcomes (including with and without censoring adjustment)? - What is the level of connectedness of evidence networks on final endpoint? |

AE, adverse event; ITC, indirect treatment comparison; VAS, visual analog scale.

Supplemental Table 3 Overview of trials identified in the SLR

| **Trial name (registry) or publication author, year** | **Treatment/comparisons assessed** | **Patients, n** | **Publications** |
| --- | --- | --- | --- |
| **Sebetralstat** | | | |
| KVD900-201 (NCT04208412) | KVD900, then placebo vs placebo, then KVD900 | 68 [1] | Aygoren-Pursun 2023^a^ [1], Aygoren-Pursun 2023^c^ [2], Smith 2022^c^ [3], Audhya 2022^c^ [4], Longhurst 2022^c^ [5], Bernstein 2023^c^ [6] |
| **C1INH-nf** | | | |
| Study: LEVP-2006-1 (NCT00438815) | C1INH-nf | 113 [7] | Riedl 2012^a^ [7], Riedl 2013 [8], Kalfus 2009^c^ [9], Lumry 2013^b^ [10] |
| LEVP-2005-1 (Part A) (NCT00289211) | C1INH-nf vs placebo | 83 [11] | Zuraw 2010^a^ [12], Hofstra 2012 [13], Lumry 2013^b^ [10] |
| LEVP- 2005-1 (Part B) (NCT01005888) | C1INH-nf vs placebo | 26 [14] | Zuraw 2010^a^ [12], Hofstra 2012 [13], Lumry 2013^b^ [10] |
| LEVP-2006-4 (NCT00462709) | C1INH-nf | 146 [15] | Lumry 2013^b^ [10] |
| **Ecallantide** | | | |
| EDEMA 3 (DX-88/14) (NCT00262080) | DX-88 (ecallantide) vs placebo | 72 [16] | Cicardi 2010^a^ [16], Bernstein 2012^b^ [17], MacGinnitie 2012^b^ [18], Banta 2011^b^ [19], Sheffer 2011^b^ [22], Riedl 2010^b^ [23], Sheffer 2013^b^ [22], Lumry 2012^b,c^ [23], MacGinnitie 2013^b^ [24], Rubinstein 2014^b^ [25] |
| EDEMA4 (DX-88/20) (NCT00457015) | Ecallantide vs placebo | 96 [26] | Levy 2010^a^ [26], Bernstein 2012^b^ [17], MacGinnitie 2012^b^ [18], Banta 2011^b^ [19], Sheffer 2011^b^ [22], Riedl 2010^b^ [23], Sheffer 2013^b^ [22], Lumry 2012^b,c^ [23], MacGinnitie 2013^b^ [24], Rubinstein 2014^b^ [25], Li 2013^b^ [27] |
| DX-88/19 (NCT00456508) | Ecallantide | 147 [28] | Lumry 2013 [28], Sheffer 2013^b^ [22], MacGinnitie 2013^b^ [24], Rubinstein 2014^b^ [25], Li 2013^b^ [27] |
| EDEMA1 | Ecallantide vs placebo | 48 [29] | Schneider 2007^a^ [29], Rubinstein 2014^b^ [25] |
| EDEMA2 (NCT01826916) | Ecallantide | 77 [30] | Sheffer 2013^b^, MacGinnitie 2013^b^ [24], Rubinstein 2014^b^ [25], Li 2013^b^ [27] |
| Li 2010 | Ecallantide vs placebo | 255 [31] | Li 2010^a,b^ [31] |
| **Icatibant** | | | |
| FAST1 (NCT00097695) | Icatibant vs placebo | 84 [32] | Cicardi 2010^a^ [33] , Malbrán 2014 [34], Maurer 2012^b,c^ [35], Farkas 2012^b,c^ [36], Farkas & Riedl 2012^b,c^ [37], Reshef 2012^b,c^ [38], Riedl 2008^b,c^ [39] |
| FAST2 (NCT00500656) | Icatibant vs tranexamic acid or placebo | 85 [40] | Cicardi 2010^a^ [33] , Bas 2013 [41], Maurer 2012^b,c^ [35], Farkas 2012^b,c^ [36], Farkas & Riedl 2012^b,c^ [37], Reshef 2012^b,c^ [38], Riedl 2008^b,c^ [39] |
| FAST3 (NCT00912093) | Icatibant vs placebo | 98 [42] | Lumry 2011^a^ [42], Lumry 2015 [43], Yang 2014^c^ [44], Li 2011^c^ [45], Maurer 2012^b,c^ [35], Farkas 2012^b,c^ [36], Farkas & Riedl 2012^b,c^ [37], Reshef 2012^b,c^ [38] |
| EASSI (NCT00997204) | Icatibant | 151 [46] | Aberer 2014 [46] |
| **Berotralstat** | | | |
| ZENITH-1 (NCT03240133;  EUCTR2016-001424-55-PL) | BCX7353 vs placebo | 58 [47] | Stobiecki 2019^c^ [47], Longhurst 2019^c^ [48] |
| **rhC1INH** | | | |
| C1-1205-01 (NCT00225147) | rhC1INH vs placebo | 77 [49] | Riedl 2013^a^ [50], Bernstein 2017^b^ [51], Baker 2018^b^ [52], Riedl 2017^b^ [53], Baker 2017^b^ [54], Zuraw 2010^b^ [55] |
| C1-1304-01 (NCT00262301) | rhC1INH vs placebo | 75 [56] | Moldovan 2012 [57], Bernstein 2017^b^ [51], Baker 2018^b^ [52], Riedl 2017^b^ [53], Baker 2017^b^ [54], Zuraw 2010^b^ [55] |
| C1-1310 (NCT01188564) | rhC1INH vs placebo | 75 [58] | Riedl 2014^a^ [59], Li 2015 [60], Li 2017 [61], Moldovan 2015^c^ [62], Bernstein 2017^b^ [51], Baker 2018^b^ [52], Riedl 2017^b^ [53], Baker 2017^b^ [54], Zuraw 2010^b^ [55] |
| Toubi 2012 | rhC1INH vs NR | 16 [63] | Toubi 2012^c^ [63] |
| Li 2013 | rhC1INH vs NR | 44 [64] | Li 2013^b,c^ [64] |
| Li 2014 | rhC1INH vs placebo | 75 [65] | Li 2014^b,c^ [65] |
| Riedl 2015 | rhC1INF vs NR | 34 [66] | Riedl 2015^b,c^ [66] |
| Baker 2017 | thC1INF vs placebo | NR | Baker 2017^b,c^ [67] |
| **pdC1INH** | | | |
| IMPACT 1 (NCT00168103) | pdC1INH vs placebo | 126 [68] | Craig 2009^a^ [68], Craig 2013^b^ [69], Schneider 2013^b^ [70] |
| IMPACT 2 (NCT00292981) | pdC1INH | 57 [71] | Bernstein 2014 [71], Craig 2012 [72], Craig 2011 [73], Craig 2013^b^ [69], Schneider 2013^b^ [70] |
| **PHA-022121 vs placebo** | | | |
| RAPIDe-1 (NCT04618211;  EUCTR2020-003445-11) | Deucrictibant v placebo | 74 [74] | Aygoren-Pursun 2023^c^ [75], Magerl 2023^c^ [76], Riedl 2023^c^ [77] |
| **C1INH concentrate vs placebo** | | | |
| Kunschak 1998 | C1INH vs placebo | 36 [78] | Kunschak 1998^c^ [78] |

C1INH, C1 esterase inhibitor; IV, intravenous; nf, nanofiltered; NR, not reported; OLE, open-label expansion; pd, plasma-derived; rh, recombinant human; SLR, systematic literature review.

^a^Primary publication.

^b^Pooled data.

^c^Conference abstract.

**Supplemental Table 4** Primary endpoint definitions and measures across trials included in the feasibility assessment

| **Author, year** | **Intervention** | **Study name** | **Primary  endpoint** | **Primary endpoint  measures** |
| --- | --- | --- | --- | --- |
| Craig et al, 2009 [68] | Berinert | IMPACT 1 | Time to onset of symptom relief | Patient responses to a standard questionnaire (not described in the publication) posed at appropriate time intervals for as long as 24 h after dosing |
| Zuraw et al, 2010 [55] | Ruconest | Pooled (C1- 1304-01 and C1-1205-01) | Time to beginning  of symptom relief | Patient-reported VAS score decrease by ≥20 mm at any location for two consecutive VAS recordings |
| Riedl et al, 2014 [59] | Ruconest | C1-1310 | Time to onset of sustained relief | TEQ Q1. How would you describe your overall HAE attack symptoms right now, compared to how you were when you took the trial medication?  Q2: Overall, has the intensity of your [relevant attack location] HAE attack symptoms begun to decrease noticeably since you received the infusion?  Time between dosing and first assessment when patient answered, “a little better,” “better,” or “much better” for Q1; answered “yes” for Q2; and had persistence of improvement at the next assessment (i.e., either the same or a better response to Q1 and “yes” to Q2), with follow-up over a 24-h period |
| Cicardi et al, 2010 [16] | Ecallantide | EDEMA3 | Median TOS 4 h after dosing | The TOS is a composite of a patient-reported response assessment for each identified symptom complex weighted based on the severity assessment at baseline. Values for the composite treatment outcome score range from +100 (designated in the protocol as significant improvement in symptoms) to −100 (significant worsening of symptoms). |
| Levy et al, 2010 [26] | Ecallantide | EDEMA4 | Change from baseline in MSCS score 4 h after dosing | The MSCS outcome score is a composite, patient-reported outcome measure based on the site or sites of symptoms, the symptom severity at baseline (1 = mild, 2 = moderate, and 3 = severe), and the response to treatment at 4 and 24 hours after dosing with patients again rating symptom severity (0 = normal, 1 = mild, 2 = moderate, and 3 = severe). |
| Cicardi et al, 2010 [32] | Icatibant | FAST-1 | Median time to clinically significant relief of the index symptom | Clinically significant symptom relief was defined as a minimum decrease in the score on the VAS of 20 to 30 mm, based on the initial symptom severity; decrease had to have been sustained for three consecutive measurements. |
| Lumry et al, 2011 [42] | Icatibant | FAST-3 | Time to 50% reduction in symptom severity | Patient-assessed time to 50% reduction from investigator baseline Global Assessment in 3-symptom composite VAS score (≥1 VAS score ≥30 mm) for cutaneous and/or abdominal attacks, maintained over three consecutive time points  Patient-assessed time to 50% reduction from investigator baseline Global Assessment in a 5-symptom composite VAS score (5-point scale; 0 for “absence of symptoms” to 4 for “very severe symptoms”) for mild-to-moderate laryngeal attacks |
| Zuraw et al, 2010 [12] | C1INH-nf | Two RCTs | Time to onset of unequivocal symptom relief at the defining site (site of the most severe symptoms) | Assessed every 15-min after initial injection, unequivocal relief defined as three consecutive reports of improvement within 4 h using the following 5-point response symptom relief scale: absent now and absent before; absent now but present before; present, symptoms new; present, symptoms worse or the same; or present, symptoms better |
| Riedl et al, 2024 [79] | Sebetralstat | KONFIDENT | Time to beginning  of symptom relief | Q. How would you describe your overall HAE attack symptoms right now, compared to how you were when you took the trial medication?  A rating of at least “a little better” on the PGI-C scale for ≥2 consecutive time points within 12 hours after first dose of study drug |

HAE, hereditary angioedema; MSCS, mean symptom complex severity; nf, nanofiltered; PGI-C, Patient Global Impression of Change; Q, question; RCT, randomized controlled trial; TEQ, Treatment Effect Questionnaire; TOS, treatment outcome score; VAS, visual analog scale.

**Supplementary Table 5** Key study design elements of trials included in the Feasibility Assessment

| **Author Year** | **Agent** | **Study name** | **Instruction to pts as when to treat (e.g., symptom onset, attack is moderate)** | **Who established baseline severity/**  **measure** | **Data censoring rules** | **Eligibility for rescue medicine** | **Rescue medications allowed** |
| --- | --- | --- | --- | --- | --- | --- | --- |
| Craig et al, 2009 [68] | pdC1INH | IMPACT 1 | Present *onsite* with an acute moderate to severe abdominal or facial attack <5 h of attack attaining moderate intensity | Pt and investigator confirmation/3-point scale [mild, moderate, severe] | Time to onset of symptom relief was censored at 24 h if received rescue medication or analgesics, antiemetics, open-label C1INH, or fresh frozen plasma after 4 h. | Eligibility for rescue medication in the first 4 h after treatment: NR  After 4 h, pts with insufficient or no symptom relief eligible for second dose of double-blind treatment (called ‘‘rescue study medication’’) | During the first 4 h after treatment: analgesics, antiemetics, open-label C1INH or fresh-frozen plasma  If insufficient or no symptom relief at 4 h: C1INH 20 U/kg in PBO arm, C1INH 10 U/kg in C1INH 10 U/kg arm, and PBO for in C1INH 20 U/kg arm |
| Zuraw et al, 2010 [55] | rhC1INH | Pooled  (C1-1304-01 and C1-1205-01) | Present *onsite* <5 h of onset of attack symptoms  Overall Severity VAS score of ≥50 mm | Pt/VAS  medical evaluation for eligibility onsite/NR | Pts who did not have beginning of relief of symptoms or minimal symptoms during the observation time were censored at the time of the last Overall VAS score for the eligible locations. | NR | NR |
| Riedl et al, 2014 [59] | rhC1INH | C1-1310 | Present *onsite* <5 h of onset of attack symptoms  Overall Severity VAS score of ≥50 mm at presentation  Just before dosing no evidence of regression of symptoms between presentation and dosing (i.e., no decrease ≥20 mm in Overall Severity VAS score) | Pt/VAS | Pts who did not achieve the beginning of persistent relief from symptoms during the 24 h assessment period were censored at their last assessment time point.  Pts who received a disallowed concomitant medication or open-label rhC1INH as rescue medication before achieving the beginning of persistent relief from symptoms were censored at the last time that the TEQ was assessed before receipt of the rescue medication or disallowed medication.  Pts who did not experience minimal symptoms during the assessment period also were censored at the last assessment time point. | 4 h after dosing, rescue medication (open-label rhC1INH 50 IU/kg up to 4200 IU) could be provided to pts who did not achieve the beginning of relief from symptoms and had a significant degree of pain, discomfort, or disability from their HAE symptoms.  At any time, at the discretion of the investigator(s), pts were allowed to receive any other rescue HAE medication.    Rescue medication also could be provided at any time to pts who experienced life-threatening, oropharyngeal-laryngeal symptoms. | Open-label rhC1INH 50 IU/kg up to a maximum of 4200 IU  Bradykinin receptor antagonists, kallikrein inhibitors, and pdC1INH prescribed at the discretion of the investigator |
| Cicardi et al, 2010 [16] | Ecallantide | EDEMA3 | Present *onsite* <8 h following onset of moderate or severe acute attacks | Pts and physician/using definitions of mild, moderate and severe, by need for treatment/  intervention and impact on ADLs | Pts not reporting the overall response as “a lot better or resolved” at 15 min through 4 h after study-drug dosing were censored at 4 h.  Pts who received additional medical intervention, including open-label ecallantide, <4 h after study-drug dosing were censored at the time of the intervention. | Pts with SUAC | For treating SUAC: CI-INH where available, fresh-frozen plasma, high dose androgens, or open-label dose of  30 mg of ecallantide  In all other cases: 5-HT3 receptor antagonists, opioids, anti-nausea medications,  C1INH |
| Levy et al, 2010 [26] | Ecallantide | EDEMA4 | Present *onsite* <8 h of onset of moderate to severe HAE attack | Using definitions of moderate and severe, by need for treatment/  intervention and impact on ADLs | For primary outcome (change from BL in MSCS) and secondary outcome (change from BL in TOS): pts were excluded from the analysis if they received a SUAC dose or if their 4-h data were missing.    For additional efficacy endpoints: pts were excluded from the analysis if they received open-label ecallantide or if their 24-h data were missing. | Pts who experienced SUAC 0 to 4 h after dosing of initial study drug, whose attacks did not improve, resolved incompletely, or relapsed 4–24 h after dosing | Single open-label dose of 30 mg of SC ecallantide |
| Cicardi et al, 2010 [32] | Icatibant | FAST-1 | Present *onsite* <6 h after an acute attack became at least moderate to undergo assessment before randomization | Pt/VAS | Pts with no documented onset of symptom relief were censored at the time of their last symptom assessment. | Double-blind phase rescue therapy for the relief of any symptom will be withheld during the period from the onset of attack for as long as possible. | C1 inhibitor concentrate, analgesics (opioids and nonopioids), antiemetics (5-HT3 receptor antagonists, antihistamines), prokinetic agents, antihistamines, and epinephrine |
| Lumry et al, 2011 [42] | Icatibant | FAST-3 | Present *onsite* <6 h after an acute attack became a least moderate (abdominal and/or cutaneous) or mild (laryngeal) in severity, and within 12 h of attack onset | Abdominal/  cutaneous attacks: pt and investigator/ Global Assessment using a VAS with ≥1 VAS score ≥30 mm  Laryngeal attack: at least mild in severity (investigator Global Assessment based on a 5-point scale; 0 for “absence of symptoms” to 4 for “very severe symptoms”) | Pts who did not achieve symptom relief within the observation period were censored at the last observation time. | Rescue medications were defined as any medications that, in the opinion of the investigator, were immediately necessary to alleviate acute symptoms from the current HAE attack. | C1INH, fresh-frozen plasma, epinephrine, and intravenous or prescription-strength nonsteroidal anti-inflammatory drugs |
| Zuraw et al, 2010 [12] | C1INH-nf | Two RCTs | Present *onsite* <4 h after onset of acute attack | Pt and investigator/ using the following scale:  • no swelling or pain  • mild swelling or pain  • moderate swelling or pain  • severe swelling or pain | Pts who did not achieve symptom relief within 4 h of the first dose were censored at 4 h.  Pt receiving narcotic rescue medication or open-label treatment with C1INH-nf due to treatment failure prior to 4 h were censored at that time. | Rescue therapy available after (1) failure to achieve significant symptom relief <4 h after the initial dosing; or (2) development of airway compromise after the initial treatment; or 3) when presenting with laryngeal angioedema | Open-label C1INH- nf; rescue with narcotics will be considered a treatment failure |
| Riedl et al, 2024 [79] | Sebetralstat | KONFIDENT | Previous attack ≥48 h before a new attack can be treated  *At home* dosing of drug as early as possible after attack onset of any location and severity at baseline | Pt/PGI-S | Time-to-event results for attacks treated using conventional OD were censored at the end of the analysis window.  Attacks were right-censored if they did not reach previously defined measure of beginning of symptom relief. | Non-laryngeal attacks: conventional on-demand treatment may be taken after the second dose of study medication  Laryngeal attacks: conventional treatment may be taken at any time after the first dose of study medication | NR |

5-HT3, 5-hydroxytryptamine 3; ADL, activity of daily living; BL, baseline; C1INH, C1 esterase inhibitor; HAE, hereditary angioedema; MSCS, mean symptom complex severity; nf, nanofiltered; NR, not reported; OD, on-demand treatment; PBO, placebo; pd, plasma-derived; PGI-C, Patient Global Impression of Change; PGI-S, Patient Global Impressions of Severity; pt patient; RCT, randomized controlled trial; rh, recombinant human; SC, subcutaneous; SOC, standard of care; SUAC, severe upper airway compromise; TEQ, Treatment Effect Questionnaire; TOS, treatment outcome score; VAS, visual analog scale.

**Supplementary Table 6** Key study design elements for safety outcomes in the Feasibility Assessment

| **Author Year** | **Agent** | **Study name** | **AE reporting and follow-up** | **AE definition** |
| --- | --- | --- | --- | --- |
| Craig et al, 2009 [68] | pdC1INH | IMPACT 1 | AEs occurring as long as 9 d after treatment were captured, SAEs as long as 12 wk after treatment. | NR |
| Zuraw et al, 2010 [55] | rhC1INH | Pooled  (C1-1304-01 and C1-1205-01) | AEs were recorded from time of administration of study medication until 90 d thereafter. | NR |
| Riedl et al, 2014 [59] | rhC1INH | C1-1310 | Safety was monitored in all patients by reports of AEs. | NR |
| Cicardi et al, 2010 [16] | Ecallantide | EDEMA3 | AEs must be reported from the enrollment (Study Day 1) through the conclusion of the follow-up visit 2. Additionally, adverse events that are suspected to be related to study procedures should be collected from the time of informed consent through enrollment and from follow-up visit 2 through follow-up visit 3. | AE: any untoward medical occurrence in a pt or clinical investigation subject administered a pharmaceutical product and that does not necessarily have a causal relationship with this treatment.    SAE: any untoward medical occurrence that results in death, is life-threatening, requires inpatient hospitalization or prolongation of existing hospitalization, results in persistent or significant disability/incapacity, or is a congenital anomaly/birth defect. |
| Levy et al, 2010 [26] | Ecallantide | EDEMA4 | AEs during or after dosing through the 7-d follow-up visit. | TEAEs defined as events with onset during or after dosing through the 7-day follow-up visit. If the presenting HAE attack resulted in hospitalization, it was captured as an SAE. |
| Cicardi et al, 2010 [32] | Icatibant | FAST-1 | AEs (including any new intercurrent illnesses or worsening of existing illnesses) will be followed up until stabilized or resolved, or until the end of the protocol-defined study period, whichever comes first.  Patients will be asked to report any AEs they may have between the study visits. | Any clinically relevant worsening of the signs and symptoms of a treated attack are considered to be AEs.  Injection site reactions not meeting the criteria of SAE do not need to be reported in addition as AEs. |
| Lumry et al, 2011 [42] | Icatibant | FAST-3 | Posttreatment | AE reporting included posttreatment worsening of symptoms. Local tolerability at the injection site was documented separately from AEs. |
| Zuraw et al, 2010 [12] | C1INH-nf | Two RCTs | From pre- to post-infusion  At 3 months after treatment. | AE: any unfavorable or unintended sign, symptom, or disease temporally associated with the use of study treatment, whether or not considered related to the study treatment  SAE: any event that results in death, is life-threatening, results in persistent or significant disability/incapacity, results in or prolongs an existing inpatient hospitalization, or is a congenital anomaly/birth defect |
| Riedl et al, 2024 [79] | Sebetralstat | KONFIDENT | Reported AEs: any TEAE, treatment-related TEAE, serious TEAE, serious treatment-related TEAE, severe TEAE, severe treatment-related TEAE, TEAE leading to permanent discontinuation, fatal TEAE | A TEAE begins on or after the first dose date of trial medication or begins before the first dose date of trial medication and increases in severity on or after the first dose date of trial medication  Serious TEAE defined as any untoward medical occurrence that at any dose results in death, is life-threatening, requires inpatient hospitalization or prolongation of existing hospitalization, results in persistent or significant disability/incapacity, is a congenital anomaly/birth defect, or is an important medical event by medical and scientific judgement |

AE, adverse event; C1INH, C1 esterase inhibitor; nf, nanofiltered; NR, not reported; RCT, randomized-controlled trial; rh, recombinant human; SAE, serious adverse event; TEAE, treatment-emergent adverse event.

**Supplemental Table 7** Deviance information criterion for time to the beginning of symptom relief

| **Stratification** | **Model** | **Deviance information criterion** |
| --- | --- | --- |
| Meta-analysis for region | Fixed effects | 4.045818 |
|  | Random effects | 4.029472 |
| Meta-analysis for sex | Fixed effects | 3.970134 |
|  | Random effects | 3.977973 |

**Supplemental Table 8** Inputs for time to the beginning of symptom relief fixed-effects model

| **Comparison** | **Time to the beginning of** **symptom relief data as reported: HR (95% CI)** | **Time to the beginning of** **symptom relief data inputs for ITC: HR (95% CI)** | **Source** |
| --- | --- | --- | --- |
| rhC1INH 50 IU/kg vs placebo |  |  |  |
| US | 1.20 (0.48–3.01) | 2.11 (1.04–4.28) | Meta-analysis for region [80] |
| Non-US | 4.82 (1.58–14.72) |  |  |
| Female | 1.22 (0.60–2.48) | 1.68 (0.91–3.07) | Meta-analysis for sex [80] |
| Male | 3.94 (1.23–12.68) |  |  |
| Sebetralstat 300 mg vs placebo | — | 2.00 (1.35–2.97) | Riedl 2024 [79] |

CI, confidence interval; HR, hazard ratio; rhC1INH, recombinant human C1 esterase inhibitor.

**Supplemental Table 9** Deviance information criterion model selection for treatment-related treatment-emergent adverse events

| **Model** | **Deviance information criterion** |
| --- | --- |
| Fixed effects | 4.244832 |
| Random effects | 4.471933 |

**Supplemental Table 10** Inputs for the treatment-related, treatment-emergent adverse event fixed-effects model

| **Study** | **Study arm** | **N** | **n** | **%** | **Odds ratio (95% CI)^a^** |
| --- | --- | --- | --- | --- | --- |
| Zuraw et al, 2010 [55] | rhC1INH 50 U/kg | 12 | 0 | 0 | 0.30 (0.01–6.32) |
| Zuraw et al, 2010 [55] | Placebo | 29 | 3 | 10 | — |
| Riedl et al, 2014 [59] | rhC1INH (50 IU/kg or 4200 IU) | 56 | 1 | 2 | 1.00 (0.04–25.63) |
| Riedl et al, 2014 [59] | Placebo | 18 | 0 | 0 | — |
| Riedl et al, 2024 [79] | Sebetralstat 300mg | 86 | 2 | 2.3 | 0.47 (0.08–2.64) |
| Riedl et al, 2024 [79] | Placebo | 83 | 4 | 4.8 | — |

CI, confidence interval; rhC1INH, recombinant human C1 esterase inhibitor.

^a^Odds ratios were calculated vs placebo. In case of 0 events, a continuity correction was applied (ie, 0.5 was added to each cell).

**Supplemental Figure 1** PRISMA flow diagram of identified trials


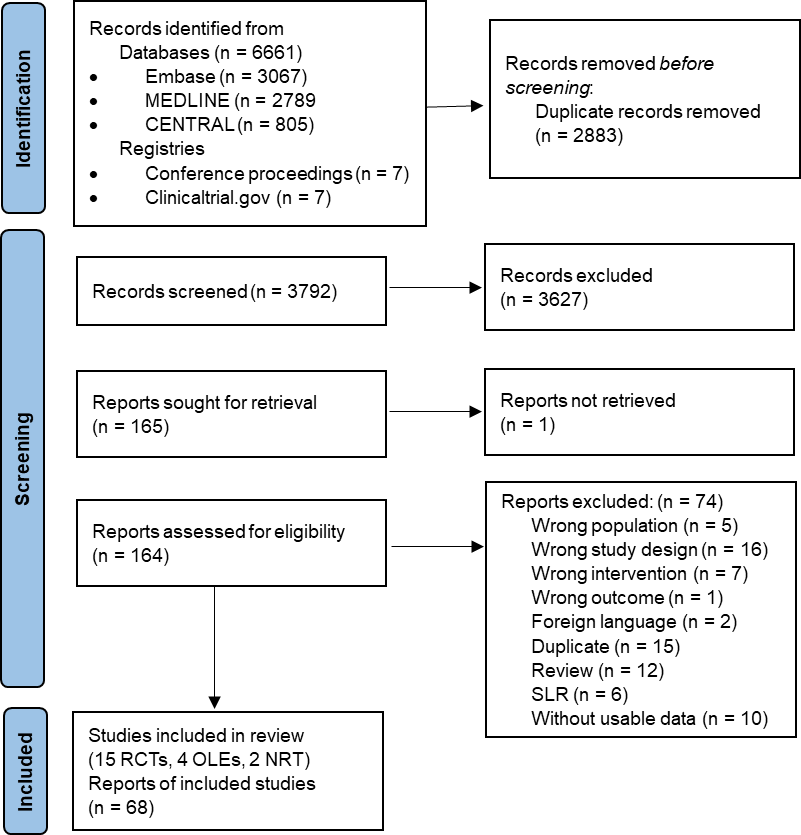


NRT, nonrandomized trial; OLE, open-label expansion; PRISMA, Preferred Reporting Items for Systematic Review and Meta-Analysis; RCT, randomized controlled trial; SLR, systematic literature review.

**Supplement References**

1. Aygören-Pürsün E, Zanichelli A, Cohn DM, et al. An investigational oral plasma kallikrein inhibitor for on-demand treatment of hereditary angioedema: a two-part, randomised, double-blind, placebo-controlled, crossover phase 2 trial. *Lancet.* 2023;401:458-469. https://doi.org/10.1016/S0140-6736(22)02406-0.
2. Aygoren-Pursun E, Zanichelli A, Cohn DM, et al. Efficacy of the oral plasma kallikrein inhibitor sebetralstat (KVD900) by attack location in a phase 2 clinical trial in patients with hereditary angioedema. Presented at: Western Society of Allergy, Asthma, and Immunology; February 5-9, 2023; Kamuela, HI. Poster 40.
3. Smith MD, Duckworth EJ, Hampton SL, Yea CM, Audhya P, Feener EP. Oral sebetralstat (KVD900) provides rapid inhibition of plasma kallikrein and fast improvement in attack symptoms in patients with hereditary angioedema. Presented at: Eastern Allergy Conference; June 2-5, 2022; Palm Beach, FL. Poster F20.
4. Audyha PA, Williams P, Yea C, Cohn D. Agreement of Patient Global Impression of Change with attack resolution or use of rescue medication in patients with hereditary angioedema. Presented at: American Academy of Allergy, Asthma & Immunology; February 25-28, 2022; Phoenix, AZ. Poster 509.
5. Longhurst HJ, Smith MD, Yea CM, Audhya PK. Sebetralstat effectiveness in the treatment of hereditary angioedema attacks rated mild or moderate at baseline in the phase 2 trial. Presented at: Australasia Society of Clinical Immunology and Allergy; August 30-September 2, 2022; Melbourne, Australia. Poster 92.
6. Bernstein JA, Riedl MA, Lumry WR, Audhya PK, Smith MD, Yea CM. Treatment with sebetralstat reduces the cumulative symptom severity of hereditary angioedema attacks in a phase 2 trial. Presented at: American Academy of Allergy, Asthma & Immunology; February 24-27, 2023; San Antonio, TX. Poster 401.
7. Riedl MA, Hurewitz DS, Levy R, Busse PJ, Fitts D, Kalfus I. Nanofiltered C1 esterase inhibitor (human) for the treatment of acute attacks of hereditary angioedema: an open-label trial. *Ann Allergy Asthma Immunol.* 2012;108:49-53. https://doi.org/10.1016/j.anai.2011.10.017.
8. Riedl MA, Lumry WR, Li HH, et al. Nanofiltered C1 esterase inhibitor for treatment of laryngeal attacks in patients with hereditary angioedema. *Am J Rhinol Allergy*. 2013;27:517-521. https://doi.org/10.2500/ajra.2013.27.3973.
9. Kalfus I, Tillotson G. Angioedema: therapeutics options to suit the pathophysiology: an update on HAE. *Chest.* 2009;136 (suppl):S1S-152S.
10. Lumry W, Manning ME, Hurewitz DS, et al. Nanofiltered C1-esterase inhibitor for the acute management and prevention of hereditary angioedema attacks due to C1-inhibitor deficiency in children. *J Pediatr.* 2013;162:1017-22.e222. https://doi.org/10.1016/j.jpeds.2012.11.030.
11. C1 esterase inhibitor (C1INH-nf) for the treatment of acute hereditary angioedema (HAE) attacks. ClinicalTrials.gov identifier: NCT01005888. Accessed September 23, 2024. https://clinicaltrials.gov/study/NCT00289211.
12. Zuraw BL, Busse PJ, White M, et al. Nanofiltered C1 inhibitor concentrate for treatment of hereditary angioedema. *N Engl J Med.* 2010b;363:513-522. https://doi.org/10.1056/NEJMoa0805538.
13. Hofstra JJ, Kleine Budde I, van Twuyver E, et al. Treatment of hereditary angioedema with nanofiltered C1-esterase inhibitor concentrate (Cetor^®^): multi-center phase II and III studies to assess pharmacokinetics, clinical efficacy and safety. *Clin Immunol.* 2012;142:280-290. https://doi.org/10.1016/j.clim.2011.11.005.
14. C1 esterase inhibitor (C1INH-nf) for the prevention of acute hereditary angioedema (HAE) attacks.ClinicalTrials.gov identifier: NCT01005888. Accessed September 23, 2024. https://clinicaltrials.gov/study/NCT01005888.
15. Open-label C1 esterase inhibitor (C1INH-nf) for the prevention of acute hereditary angioedema (HAE) attacks (CHANGE 3). ClinicalTrials.gov identifier: NCT00462709. Accessed September 23, 2024. https://clinicaltrials.gov/study/NCT00462709.
16. Cicardi M, Levy RJ, McNeil DL, et al. Ecallantide for the treatment of acute attacks in hereditary angioedema. *N Engl J Med.* 2010;363(6):523-531. https://doi.org/10.1056/NEJMoa0905079.
17. Bernstein JA, Moellman JJ. Progress in the emergency management of hereditary angioedema: focus on new treatment options in the United States. *Postgrad Med.* 2012;124:91-100. https://doi.org/10.3810/pgm.2012.05.2552.
18. MacGinnitie AJ, Campion M, Stolz LE, Pullman WE. Ecallantide for treatment of acute hereditary angioedema attacks: analysis of efficacy by patient characteristics. *Allergy Asthma Proc.* 2012;33:178-185. https://doi.org/10.2500/aap.2012.33.3528.
19. Banta E, Horn P, Craig TJ. Response to ecallantide treatment of acute attacks of hereditary angioedema based on time to intervention: results from the EDEMA clinical trials. *Allergy Asthma Proc.* 2011;32:319-324. https://doi.org/10.2500/aap.2011.32.3440.
20. Sheffer AL, Campion M, Levy RJ, Li HH, Horn PT, Pullman WE. Ecallantide (DX-88) for acute hereditary angioedema attacks: integrated analysis of 2 double-blind, phase 3 studies. *J Allergy Clin Immunol.* 2011;128:153-159.e4. https://doi.org/10.1016/j.jaci.2011.03.006.
21. Riedl M, Campion M, Horn PT, Pullman WE. Response time for ecallantide treatment of acute hereditary angioedema attacks. *Ann Allergy Asthma Immunol.* 2010;105:430-436.e2. https://doi.org/10.1016/j.anai.2010.09.005.
22. Sheffer AL, MacGinnitie AJ, Campion M, Stolz LE, Pullman WE. Outcomes after ecallantide treatment of laryngeal hereditary angioedema attacks. *J Ann Allergy Asthma Immunol.* 2013;110:184-188.E2. http://dx.doi.org/10.1016/j.anai.2012.12.007.
23. Lumry WR, Li HH, MacGinnitie AJ, et al. Efficacy and safety of ecallantide treatment for HAE attack in patients treated with both ecallantide and placebo. Presented at: American Academy of Allergy, Asthma & Immunology; March 2-6, 2012; Orlando, FL. Abstract 829.
24. MacGinnitie AJ, Davis-Lorton M, Stolz LE, Tachdjian R. Use of ecallantide in pediatric hereditary angioedema*. Pediatrics.* 2013;132:e490-e497. https://doi.org/10.1542/peds.2013-064.
25. Rubinstein E, Stolz LE, Sheffer AL, Stevens C, Bousvaros A. Abdominal attacks and treatment in hereditary angioedema with C1-inhibitor deficiency. *BMC Gastroenterol.* 2014;14:71. https://doi.org/10.1186/1471-230X-14-71.
26. Levy RJ, Lumry WR, McNeil DL, et al. EDEMA4: a phase 3, double-blind study of subcutaneous ecallantide treatment for acute attacks of hereditary angioedema. *Ann Allergy Asthma Immunol.* 2010;104:523-529. https://doi.org/10.1016/j.anai.2010.04.012.
27. Li HH, Campion M, Craig TJ, et al. Analysis of hereditary angioedema attacks requiring a second dose of ecallantide. *Ann Allergy Asthma Immunol.* 2013;110:168-172. https://doi.org/10.1016/j.anai.2012.12.004.
28. Lumry WR, Bernstein JA, Li HH, et al. Efficacy and safety of ecallantide in treatment of recurrent attacks of hereditary angioedema: open-label continuation study. *Allergy Asthma Proc.* 2013;34:155-161. https://doi.org/10.2500/aap.2013.34.3653.
29. Schneider L, Lumry W, Vegh A, Williams AH, Schmalbach T. Critical role of kallikrein in hereditary angioedema pathogenesis: a clinical trial of ecallantide, a novel kallikrein inhibitor. *J Allergy Clin Immunol.* 2007;120:416-422. https://doi.org/10.1016/j.jaci.2007.04.028.
30. Subcutaneous treatment with icatibant for acute attacks of hereditary angioedema. ClinicalTrials.gov identifier: NCT01826916. Updated . Accessed September 23, 2024. https://clinicaltrials.gov/study/NCT01826916.
31. Li HH, Horn PT, Pullman WE. Overview of anaphylaxis following ecallantide treatment for acute attacks of hereditary angioedema. Poster presented at: International Drug Hypersensitivity Meeting; April 22-25, 2010; Rome, Italy.
32. Subcutaneous treatment with icatibant for acute attacks of hereditary angioedema.ClinicalTrials.gov identifier: NCT00097695. Accessed September 23, 2024. <https://clinicaltrials.gov/study/NCT00097695>.
33. Cicardi M, Banerji A, Bracho F, et al. Icatibant, a new bradykinin-receptor antagonist, in hereditary angioedema. *N Engl J Med.* 2010b;363:532-541. https://doi.org/10.1056/NEJMoa0906393.
34. Malbrán A, Riedl M, Ritchie B, et al. Repeat treatment of acute hereditary angioedema attacks with open-label icatibant in the FAST-1 trial. *Clin Exp Immunol.* 2014;177:544-553. https://doi.org/10.1111/cei.12358.
35. Maurer M, Reshef A, Craig T, Cicardi M. Icatibant effectively alleviates non-laryngeal attacks of hereditary angioedema type I and II irrespective of attack severity or historical attack frequency. Presented at: European Academy of Allergy and Clinical Immunology. June 16-20, 2012; Geneva, Switzerland.
36. Farkas H, Katelaris C, Riedl M, Kivity S. Integrated results from three phase III trials to explore the efficacy of icatibant in non-laryngeal attacks of type I and II hereditary angioedema. Presented at: European Academy of Allergy and Clinical Immunology; June 16-20, 2012; Geneva, Switzerland. Abstract 195.
37. Farkas H, Riedl M. Efficacy of icatibant in non-laryngeal attacks of type I and II hereditary angioedema: integrated results from three phase 3 trials. American Academy of Allergy, Asthma & Immunology; March 2-6, 2012; Orlando, FL. Abstract 820.
38. Reshef A, Levy R, Craig T. Efficacy of icatibant is consistent by attack frequency and baseline severity in the treatment of type I and II hereditary angioedema (HAE) attacks. *J Allergy Clin Immunol.* 2012;129(suppl, abstr 822):AB218. https://doi.org/10.1016/j.jaci.2011.12.100.
39. Riedl M. Icatibant, a selective bradykinin B2 receptor antagonist, proves effective and safe in treating the symptoms of hereditary angioedema (HAE) attacks. *J Allergy Clin Immunol.* 2008;121(suppl 1, abstr 398):S103.
40. Subcutaneous treatment with icatibant for acute attacks of hereditary angioedema (HAE) (FAST2).ClinicalTrials.gov identifier: NCT005000656. Accessed September 23, 2024. <https://clinicaltrials.gov/study/NCT00500656>.
41. Baş M, Greve J, Hoffmann TK, et al. Repeat treatment with icatibant for multiple hereditary angioedema attacks: FAST-2 open-label study. *Allergy.* 2013;68:1452-1459. https://doi.org/10.1111/all.12244.
42. Lumry WR, Li HH, Levy RJ, et al. Randomized placebo-controlled trial of the bradykinin B2 receptor antagonist icatibant for the treatment of acute attacks of hereditary angioedema: the FAST-3 trial. *Ann Allergy Asthma Immunol.* 2011;107:529-537. https://doi.org/10.1016/j.anai.2011.08.015.
43. Lumry WR, Farkas H, Moldovan D, et al. Icatibant for multiple hereditary angioedema attacks across the controlled and open-label extension phases of FAST-3. *Int Arch Allergy Immunol.* 2015;168:44-55. https://doi.org/10.1159/000441060.
44. Yang W, Hebert J, Ritchie B, et al. Analysis of icatibant for the treatment of laryngeal hereditary angioedema attacks in the FAST-3 study. Presented at: Canadian Society of Allergy and Clinical Immunology and AllerGen Abstracts. *Allergy Asthma Clin Immunol*. 2014;10(suppl 2, abstr):A51. https://doi.org/10.1186/1710-1492-10-S2-A51.
45. Li HH, Craig T. Efficacy of icatibant persists over 8 h in patients with hereditary angioedema: results from a phase III study. Presented at: American College of Allergy, Asthma and Immunology. November 3-8, 2011; Boston, MA. Abstract 113.
46. Aberer W, Maurer M, Reshef A, et al. Open-label, multicenter study of self-administered icatibant for attacks of hereditary angioedema. *Allergy.* 2014;69:305-314. HttpsS://doi.org/10.1111/all.12303.
47. Stobiecki M, Aygören-Pürsün E, Andarawewa S, et al. Clinical evaluation of pharmacokinetics, pharmacodynamics, safety, and efficacy dose-response of BCX7353 as an acute treatment for angioedema in patients with hereditary angioedema (HAE). Presented at: C1-inhibitor Deficiency & Angioedema Workshop; May 23-26, 2019; Budapest, Hungary. Abstract O24.
48. Longhurst H, Moldovan D, Bygum A, et al. Oral plasma kallikrein inhibitor BCX7353 is safe and effective as an on-demand treatment of angioedema attacks in hereditary angioedema (HAE) patients: results of the ZENITH-1 Trial. Presented at: American Academy of Allergy, Asthma & Immunology; February 22-25, 2019; San Francisco, CA. Abstract 110.
49. Pharming Technologies B.V. Recombinant human C1 inhibitor for the treatment of acute attacks in patients with hereditary angioedema. ClinicalTrials.gov identifier: NCT00225147. Accessed September 23, 2024. <https://clinicaltrials.gov/study/NCT00225147>.
50. Riedl MA, Levy RJ, Suez D, et al. Efficacy and safety of recombinant C1 inhibitor for the treatment of hereditary angioedema attacks: a North American open-label study. *Ann Allergy Asthma Immunol.* 2013;110:295-299. https://doi.org/10.1016/j.anai.2013.02.007.
51. Bernstein JA, Relan A, Harper JR, Riedl M. Sustained response of recombinant human C1 esterase inhibitor for acute treatment of hereditary angioedema attacks. *Ann Allergy Asthma Immunol.* 2017;118:452-455. https://doi.org/10.1016/j.anai.2017.01.029.
52. Baker JW, Bernstein JA, Harper JR, Relan A, Riedl MA. Efficacy of recombinant human C1 esterase inhibitor across anatomic locations in acute hereditary angioedema attacks. *Allergy Asthma Proc.* 2018;39:359-364. https://doi.org/10.2500/aap.2018.39.4151.
53. Riedl MA, Li HH, Cicardi M, Harper JR, Relan A. Recombinant human C1 esterase inhibitor for acute hereditary angioedema attacks with upper airway involvement. *Allergy Asthma Proc.* 2017;38:462-466. https://doi.org/10.2500/aap.2017.38.4090.
54. Baker JW, Reshef A, Moldovan D, Harper JR, Relan A, Riedl MA. Recombinant human C1-esterase inhibitor to treat acute hereditary angioedema Attacks in adolescents. *J Allergy Clin Immunol Pract.* 2017;5:1091-1097. https://doi.org/10.1016/j.jaip.2016.11.005.
55. Zuraw B, Cicardi M, Levy RJ, et al. Recombinant human C1-inhibitor for the treatment of acute angioedema attacks in patients with hereditary angioedema. *J Allergy Clin Immunol.* 2010a;126:821-827. https://doi.org/10.1016/j.jaci.2010.07.021.
56. Recombinant human C1 inhibitor for the treatment of acute attacks in patients with hereditary angioedema. ClinicalTrials.gov identifier: NCT00262301. Accessed September 23, 2024.<https://clinicaltrials.gov/study/NCT00262301>.
57. Moldovan D, Reshef A, Fabiani J, et al. Efficacy and safety of recombinant human C1-inhibitor for the treatment of attacks of hereditary angioedema: European open-label extension study. *Clin Exp Allergy*. 2012;42:929-935. https://doi.org/:10.1111/j.1365-2222.2012.03984.x.
58. Efficacy, safety and immunogenicity study of recombinant human C1 inhibitor for the treatment of acute HAE attacks. ClinicalTrials.gov identifier: NCT01188564. Accessed September 23, 2024. <https://clinicaltrials.gov/study/NCT01188564>.
59. Riedl MA, Bernstein JA, Li H, et al. Recombinant human C1-esterase inhibitor relieves symptoms of hereditary angioedema attacks: phase 3, randomized, placebo-controlled trial. *Ann Allergy Asthma Immunol.* 2014;112:163-169.e1. https://doi.org/10.1016/j.anai.2013.12.004.
60. Li HH, Moldovan D, Bernstein JA, et al. Recombinant human-C1 inhibitor is effective and safe for repeat hereditary angioedema attacks. *J Allergy Clin Immunol Pract.* 2015;3:417-423. https://doi.org/10.1016/j.jaip.2014.12.013.
61. Li HH, Reshef A, Baker JW, Harper JR, Relan A. Efficacy of recombinant human C1 esterase inhibitor for the treatment of severe hereditary angioedema attacks. *Allergy Asthma Proc.* 2017;38:456-461. https://doi.org/10.2500/aap.2017.38.408.
62. Moldovan D, Bernstein JA, Cicardi M. Recombinant replacement therapy for hereditary angioedema due to C1 inhibitor deficiency. *Immunotherapy.* 2015;7:739-752. https://doi.org/10.2217/imt.15.44.
63. Toubi E, Baker J, Moldovan D, Levy R, Relan A. Safety and efficacy evaluation of rhC1INH for the treatment of hereditary angioedema attacks in adolescent patients. Presented at: European Academy of Allergy & Clinical Immunology; June 16-20, 2012; Geneva, Switzerland. Abstract 199.
64. Li H, Moldovan D, Bernstein J, et al. Efficacy and safety of recombinant human C1 esterase inhibitor for acute attacks of hereditary angioedema: an open-label study. Presented at: American College of Allergy, Asthma and Immunology. November 7-11, 2013; Baltimore, MD. A92-A93.
65. Li H, Reshef A, Farkas H, et al. Efficacy of recombinant human C1 inhibitor for the treatment of hereditary angioedema patients with severe attacks. Presented at: American College of Allergy, Asthma and Immunology. November 6-10, 2014; Atlanta, GA. A5.
66. Riedl M, Cicardi M. Efficacy of recombinant C1 inhibitor for laryngeal attacks of hereditary angioedema. Presented at: European Academy of Allergy and Clinical Immunology; June 6-10, 2015; Barcelona, Spain. Abstract 470.
67. Baker JW, Bernstein JA, Harper JR, Relan A, Riedl MA. Efficacy of recombinant human C1 esterase inhibitor (rhC1INH) across anatomical locations in acute hereditary angioedema (HAE) *J Allergy Clin Immunol.* 2017;139:AB235.
68. Craig TJ, Levy RJ, Wasserman RL, et al. Efficacy of human C1 esterase inhibitor concentrate compared with placebo in acute hereditary angioedema attacks. *J Allergy Clin Immunol.* 2009;124:801-808. https://doi.org/10.1016/j.jaci.2009.07.017.
69. Craig TJ, Rojavin MA, Machnig T, Keinecke HO, Bernstein JA. Effect of time to treatment on response to C1 esterase inhibitor concentrate for hereditary angioedema attacks. *Ann Allergy Asthma Immunol*. 2013;111:211-215. https://doi.org/10.1016/j.anai.2013.06.021.
70. Schneider L, Hurewitz D, Wasserman R, et al. C1-INH concentrate for treatment of acute hereditary angioedema: a pediatric cohort from the I.M.P.A.C.T. studies. *Pediatr Allergy Immunol.* 2013;24:54-60. https://doi.org/10.1111/pai.12024.
71. Bernstein JA, Machnig T, Keinecke HO, Whelan GJ, Craig TJ. The effect of weight on the efficacy and safety of C1 esterase inhibitor concentrate for the treatment of acute hereditary *Clin Ther.* 2014;36:518-525. https://doi.org/10.1016/j.clinthera.2014.02.005.
72. Craig TJ, Bewtra AK, Hurewitz D, et al. Treatment response after repeated administration of C1 esterase inhibitor for successive acute hereditary angioedema attacks. *Allergy Asthma Proc.* 2012;33:354-361. https://doi.org/10.2500/aap.2012.33.3589.
73. Craig TJ, Bewtra AK, Bahna SL, et al. C1 esterase inhibitor concentrate in 1085 Hereditary Angioedema attacks--final results of the I.M.P.A.C.T.2 study. *Allergy.* 2011;66:1604-1611. https://doi.org/10.1111/j.1398-9995.2011.02702.x.
74. Dose-ranging study of oral PHA-022121 for acute treatment of angioedema attacks in patients with hereditary angioedema (RAPIDe-1). ClinicalTrials.gov identifier: NCT04618211. Accessed September 23, 2024. <https://clinicaltrials.gov/study/NCT04618211>.
75. Aygoren-Pursun E, Anderson J, Baez ML, et al. Treatment with oral administered bradykinin B2 receptor inhibitor PHVS416 improves hereditary angioedema attack symptoms. Presented at: European Academy of Allergy and Clinical Immunology Hybrid Congress, June 9-11, 2023; Hamburg, Germany. ﻿Abstract 001557.
76. Magerl M, Anderson J, Aygören-Pürsün E, et al. Efficacy and safety of the oral bradykinin B2 receptor antagonist deucrictibant immediate release capsule (PHVS416) in treatment of hereditary angioedema attacks: topline results of RAPIDe 1 Phase 2 Trial. Presented at: HAEi Regional Conference EMEA; September 1-3, 2023; Munich, Germany.
77. Riedl M, Aygören-Pürsün E, Cohn DM et al. Deucrictubant immediate-release capsule reduces time to end of progression of hereditary angioedema attacks’ manifestations. *Ann Allergy Asthma Immunol.* 2023;131:S15-S94. Abstract P081.
78. Kunschak M, Engl W, Maritsch F, et al. A randomized, controlled trial to study the efficacy and safety of C1 inhibitor concentrate in treating hereditary angioedema. *Transfusion*. 1998;38:540-549. https://doi.org/10.1046/j.1537-2995.1998.38698326333.x.
79. Riedl MA, Farkas H, Aygören-Pürsün E, et al. Oral sebetralstat for on-demand treatment of hereditary angioedema attacks. *N Engl J Med.* 2024;391:32-43. https://doi.org/10.1056/NEJMoa2314192.
80. RUCONEST (C1 esterase inhibitor [recombinant]) Prescribing information. Pharming Healthcare Inc; April 2020.
